# MOGSA: Integrative Single Sample Gene-set Analysis of Multiple Omics Data*[Fn FN2]

**DOI:** 10.1074/mcp.TIR118.001251

**Published:** 2019-06-26

**Authors:** Chen Meng, Azfar Basunia, Bjoern Peters, Amin Moghaddas Gholami, Bernhard Kuster, Aedín C. Culhane

**Affiliations:** ‡Chair of Proteomics and Bioanalytics, Technische Universität München, Freising, Germany; §Bavarian Biomolecular Mass Spectrometry Center (BayBioMS), TUM, Freising, Germany; ¶Department of Data Science, Division of Biostatistics and Computational Biology, Dana-Farber Cancer Institute, Boston, Massachusetts 02215; ‖La Jolla Institute for Allergy and Immunology, 9420 Athena Circle, La Jolla, California 92037; **Department of Biostatistics, Harvard T.H. Chan School of Public Health, Boston, Massachusetts 02215

**Keywords:** Computational Biology, Bioinformatics software, Mass Spectrometry, RNA SEQ, Metabolomics, gene set analysis, Multi-omics integration, single sample, tumor subtype

## Abstract

Gene-set analysis (GSA) summarizes individual molecular measurements to more interpretable pathways or gene-sets and has become an indispensable step in the interpretation of large-scale omics data. However, GSA methods are limited to the analysis of single omics data. Here, we introduce a new computation method termed multi-omics gene-set analysis (MOGSA), a multivariate single sample gene-set analysis method that integrates multiple experimental and molecular data types measured over the same set of samples. The method learns a low dimensional representation of most variant correlated features (genes, proteins, etc.) across multiple omics data sets, transforms the features onto the same scale and calculates an integrated gene-set score from the most informative features in each data type. MOGSA does not require filtering data to the intersection of features (gene IDs), therefore, all molecular features, including those that lack annotation may be included in the analysis. Using simulated data, we demonstrate that integrating multiple diverse sources of molecular data increases the power to discover subtle changes in gene-sets and may reduce the impact of unreliable information in any single data type. Using real experimental data, we demonstrate three use-cases of MOGSA. First, we show how to remove a source of noise (technical or biological) in integrative MOGSA of NCI60 transcriptome and proteome data. Second, we apply MOGSA to discover similarities and differences in mRNA, protein and phosphorylation profiles of a small study of stem cell lines and assess the influence of each data type or feature on the total gene-set score. Finally, we apply MOGSA to cluster analysis and show that three molecular subtypes are robustly discovered when copy number variation and mRNA data of 308 bladder cancers from The Cancer Genome Atlas are integrated using MOGSA. MOGSA is available in the Bioconductor R package “mogsa.”

## Abstract (precis)

Gene-set analysis (GSA) summarizes information from molecule to gene-set level to facilitate biological interpretation of molecular profiling experiments. We present a statistical framework for single sample GSA of multiple 'omic data. MOGSA learns the most variant biomolecules and integrates data sets to generate gene set scores (GSS) for each sample. It extracts the contribution of individual data sets and biomolecules to GSS. MOGSA is rigorously benchmarked with simulated and real biological data and is implemented in the Bioconductor package “mogsa.”

## Graphical Abstract

**Figure:**
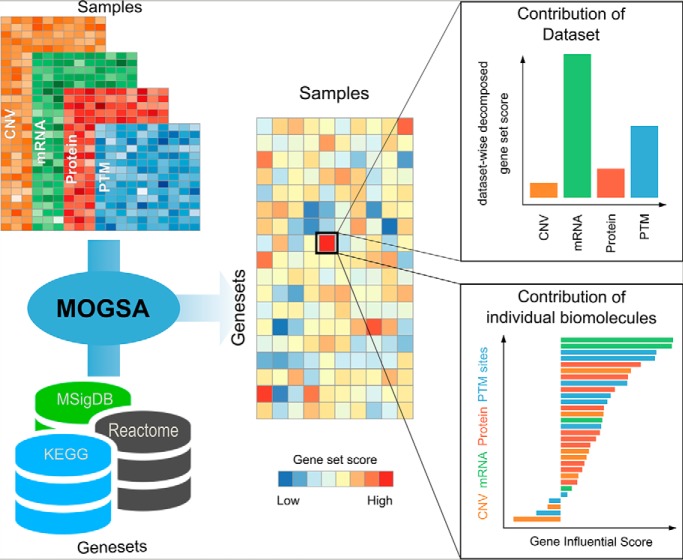

**Highlights**

- MOGSA is a new integrative multi 'omics single sample gene set analysis method.
- Integrating diverse biomolecular data increases the power to discover gene sets.
- MOGSA evaluates the contribution of biomolecules, samples and data sets to gene sets.
- The method and tutorial are available in the R/Bioconductor package mogsa.

Increasing numbers of studies report comprehensive molecular profiling using multiple different experimental approaches on the same set of biological samples. These multi-omics studies can potentially yield great insights into the complex molecular machinery of biological systems. High-throughput sequencing allows quantification of global DNA variation and whole transcriptome RNA expression (1, 2). Mass spectrometry (MS)-based proteomics can identify and quantify most proteins expressed in human tissues or cell lines (3). Emerging single cell sequencing technologies enable simultaneous measurement of transcriptomes and protein markers expressed in the same cell, using CITE-seq or REAP-seq (4, 5). Integrating, interpreting and generating biological hypothesis from such complex data sets is a considerable challenge.

Gene-set analysis (GSA)^1^ is widely used in the analysis of genome scale data and is often the first step in the biological interpretation of lists of genes or proteins that are differentially expressed between phenotypically distinct groups (6). These methods use external biological information, including gene ontologies, to reduce thousands of genes or proteins into lists of gene-sets that describe cellular pathways, subcellular localization, transcription factors or miRNA targets etc., thus facilitating hypothesis generation.

Large scale omics studies or single cell studies may have limited *a priori* knowledge of phenotype groups or may aim to discover new molecular subtypes in a panel of experimental conditions or tissues with complex phenotypes, exemplified by The Cancer Genome Atlas (TCGA) (7) and the Clinical Proteomic Tumor Analysis Consortium (CPTAC) (8). Classical GSA methods that require phenotypically distinct groups (6) have limited application in such cases and several unsupervised, single sample GSA (ssGSA) methods have been developed (9–12). These methods do not require prior availability of phenotypic or clinical data. Arguably, one of the most popular approaches is single-sample GSEA that ranks genes according to the empirical cumulative distribution function and calculates a single sample-wise gene-set score by comparing the scores of genes that are inside and outside a gene-set (10). A related method, gene-set variation analysis (GSVA), also calculates sample-wise gene-set enrichment as a function of the genes that are inside and outside a gene-set, and also uses a similar Kolmogorov-Smirnov-like rank statistic to assess the enrichment score, but genes are ranked using a kernel estimation of a cumulative density function (9). These single-sample GSA methods are designed for the analysis of a single data set, and do not integrate or calculate a single sample GSA score on multiple data sets simultaneously.

Here, we present a novel unsupervised single-sample gene-set analysis that calculates an integrated enrichment score using all the information in multiple omics data sets, named “multi-omics GSA” (MOGSA). The method relies on matrix factorization (MF), powerful methods that can be used to learn patterns of biological significance in high dimensional data (13) as well as identify and exclude batch effects (14). Coupled or tensor MF methods can learn latent correlated structure within and between omics data sets (15–18) and have been applied to the analysis of molecular data from different technology platforms (19) and integration of diverse multi-omics data (15, 17).

An attractive characteristic of coupled or tensor MF approaches, is that they identify global correlated patterns among samples or observations. They can be applied to integrating data from experimental platforms that include known and unknown molecules (for example lipidomics, metabolomics) or molecules that are difficult to map one-to-one between data sets (*e.g.* transcript variants to proteins). Therefore, these approaches do not require pre-filtering of gene identifiers in each data set to a common intersecting subset of features. Although coupled MF or latent factor methods are powerful, they identify components, the interpretation of which, can be challenging and may require domain knowledge (15, 20–22). To solve this problem, MOGSA incorporates gene-set annotation in the correlated patterns of molecules resulting from MF, calculates scores for gene-sets in each biological sample, providing simple, accessible biological interpretation. We showed that integrative ssGSA by MOGSA has higher sensitivity and specificity for the detection of differentially expressed gene-sets compared with popular ssGSA approaches when applied to simulated data. To demonstrate result interpretation and application, we applied MOGSA to both small- and large-scale biological data from high throughput experiments.

## EXPERIMENTAL PROCEDURES

### 

#### 

##### Experimental Design and Statistical Rationale

This project describes a new algorithm called MOGSA. The mathematical details of the algorithm are provided in the Supplementary Methods. We rigorously tested and validated the performance of the method using four distinct use-cases. First, we used simulated data to benchmark the performance of MOGSA and compared it to the most widely used methods in the field. Using diverse scenarios, each with 100 simulated data sets, we demonstrated that MOGSA can integrate multiple data sets thus increasing the sensitivity to identify gene-sets with subtle perturbations. Second, using well-characterized multi-omics cell line data, we demonstrate the benefit of removing a source of noise (batch effect/biological bias) by excluding a component to amplify the signal in gene-set analysis. We applied this to removing the effect of cell line doubling time in multiple transcriptomics data sets of 59 cell lines. The third use-case examined one of most common needs of biological laboratories; the integrative analysis of diverse molecular data obtained on a small number of biological samples. We integrated transcriptome, proteome and phosphoproteome data on four iPS ES cell lines and demonstrated how to interpret the gene-set scores to reveal which data set contributed most to a specific biology process. Finally, the fourth use-case examined molecular subtype discovery using multi-omics data by the integrative analysis of copy number variation and transcriptome data of 308 bladder tumors. We demonstrate how to rigorously apply the multi-omics single sample GSA method to discover molecular subtypes and interpret the biological basis for each tumor subtype in a large-scale multi-omics studies. In each case, the data are publicly available, and we provide details on the methods and code such that our analysis can be reproduced.

##### Data Simulation

We simulated 100 multiple omics data projects. Each simulated data set was a triplet (*K* = 3) containing three data matrices (supplemental Fig. S1), each matrix had the dimension 1000 × 30, representing 30 matched observations (*n* = 30) and 1,000 features (*p_k_* = 1000). Each data set had an annotation matrix, which assigned each feature to one of 20 “gene-sets.” The gene-sets were set to be either overlapping or non-overlapping with each other. In the nonoverlap setting, there were no shared candidate features in different gene-sets and therefore, the exact number of DE genes could be exactly controlled. In the overlap setting, the candidate features of different gene-sets were randomly selected and thus one feature may belong to more than one gene-set. As a result, the exact number of DE features was not precise, but this scenario is more analogous to real gene-set annotations. The binary annotation matrix had dimensions of 1000 features × 20 gene-sets. Each gene-set contained 50 genes. The 30 observations were defined by 6 equal sized clusters with 5 samples per cluster.

In each observation, 5 out of 20 gene-sets were simulated as differentially expressed (DE). For observations in the same cluster, the same set of DE gene-sets were randomly selected as we assumed that differentially expressed (DE) gene-sets define the difference between clusters. For a DE gene-set, several genes was randomly simulated as DE genes (DEG), denoted as DEG_j_. Random selection of DEGs means that the DEGs in different data sets may overlap. In separate simulations, we varied the number of DEGs per gene-set (*e.g.* 5, 10, and 25 out of 50) or mean signal to noise ratio.

We used the following linear additive model adapted from (9), the expression or abundance of gene on *i*th row and *j*th column is simulated as

(1) $$X_{ij}=\alpha_{i}+\beta_{l}+\gamma_{ij}+\varepsilon_{ij}$$

where with *i* = *1*, …, *p* is a gene specific effect. β*_l_* ∼ N(μ = 0, σ = *s*) is the cluster effect. For observations belonging to the same cluster *l*, the same β*_l_* was applied. The cluster effect factor (categorical variable) was introduced following the hypothesis that observations from the same clusters are driven by some common pathways or “gene-sets” and ensures that observations from the same cluster have a higher within than between cluster correlation. The six correlated clusters in the simulated data were captured by the first five components. The cluster effect β*_l_* ∼ N(μ = 0, σ = *s*) was sampled from a distribution with a mean of 0 and standard deviation *s.* The standard deviation (*s*) adjusts the correlation between observations in the same cluster, and thus each cluster can have different within cluster variance and different proportions of variance would be captured by the top five components. In this study, we set s = 0.3, 0.5 and 1.0, which led to 25%, 30 and 50% of total variance captured by the top 5 components. ε ∼ N(μ = 0, σ = 1) is the noise factor. γ_ij_ is the differential expression factor describing if a gene is differentially expressed (DE):

(2) $$\gamma_{ij}\{\begin{array}{cc} ∼\text{N}\left(\mu =m,\sigma =1\right) & if\;i\in DEG_{j}\\ =0 & otherwise \end{array}$$

Apart from the retained variance by top five components, two other parameters were tuned in the simulation study. First, the number of DEGs in a DE gene-set (5, 10, and 25 out of 50 DEGs). The second parameter was different signal-to-noise ratios, which was tuned through modifying *m* in expression (2). The candidate values of *m* were 0.3, 0.5, and 0.8 standing for low, medium and high signal-to-noise ratio. In total, 100 projects of triplet data sets were generated. The three matrix triplets were analyzed by MOGSA. NMM, GSVA and ssGSEA, only accept one matrix as input; therefore, the three simulated matrices were concatenated into one grand matrix in these analyses. The performance was assessed by the area under the ROC curve (AUC).

##### Downloading and Processing of NCI60 Cancer Data

Processed mRNA expression data (normalized score averaged from 5 microarray platform) and clinical information were downloaded from CELLMINER (download date: 06/01/2017) (23). Quantitative proteome profiles were downloaded from the supplementary table of (24). The proteome data were quantified and normalized using the iBAQ method (25) and iBAQ values were transformed by x_i_ = log10(iBAQ_i_ + 1). The LC cell line SNB19 and melanoma cell line MDAN were missing mRNA data and were therefore excluded from the analysis.

##### Determining the Number of Components that Capture the Correlated Structure Between NCI60 Transcriptome and Proteome Data

A random sampling method was applied to determine the number of components that represented significant correlated structure between data sets. First, MFA was applied to NCI60 transcriptome and proteome data and the (true) variance associated with each MFA component was recorded. Next, cell line labels were randomly shuffled in both transcriptomics and proteomics data and the variance of components were calculated from the randomly labels data. We repeated this process 20 times to estimate the null distribution of variances associated with each component. The variance of the top three components was significantly higher than the null distribution (supplemental Fig. S2).

##### Downloading and Processing of the iPS ES 4-Plex Data

The transcriptomics (RNA-sequencing), proteomics and phoshphoproteomics data were downloaded from Stem Cell-Omic Repository (supplemental Table S1, S2, and S5 from http://scor.chem.wisc.edu/data.php) (26). In this study, we used the 4-plex data, which consisted of 17,347 genes, 7952 proteins and 10,499 sites of phosphorylation in four cell lines. For the transcriptomics data, the expression levels of genes were represented by RPKM values. Raw RPKM and MS-based proteomics intensity data may quantify many low abundance molecules, therefore, is strongly skewed and contain outliers (27). However, MFA, the matrix decomposition method used in MOGSA, is sensitive to outliers, because it minimizes sum squared error between original matrix and its low-rank approximation. Therefore, low abundance genes with a mean RPKM (across the 12 samples) lower than 1 were excluded and RPKM values were further log transformed (log10). The distribution of log transformed data before and after filtering is shown in supplemental Fig. S3. The mean RPKM value of the three replicates was used. When a gene symbol was present more than once in a data set, the one with higher average RPKM was retained. The iTRAQ quantification of protein and phosphorylation sites was performed by TagQuant (28), as described in (26). The intensities of protein and phospho-sites were log transformed (base 10). Protein and phospho-sites with low intensity (summed log intensity across samples < 20) were removed to exclude low abundant proteins/sites that are detected in a small number of samples. In this data set, we observed that an intensity threshold of 20 was optimal to retain proteins/sites that were consistently measured in all three replicates of one of the four cell types (average 6.7 in each replicate; see supplemental Fig. S3) but missed in other cell types. Therefore, proteins/sites exclusive to a cell type, which may represent interesting biology, were retained. In the proteomics data, proteins that were not mapped to an official gene symbol were removed. After filtering, 10,961, 5817, and 7912 features were retained in the transcriptomic, proteomic and phospho-proteomic data sets. A few missing values were still present and replaced with zero values. The enrichment analysis was performed on the gene symbol levels, the specific phosphorylation sites were not considered. PCA of each individual data set is shown in supplemental Fig. S4. The strongest signal (first PCs) in all three data sets was the difference between NFF cells and the stem cell lines, and this difference was particularly apparent in the proteomics data sets. The second and third components represented subtle differences between iPSC and ESC lines.

##### Downloading and Processing of TCGA Bladder Cancer Data

Normalized Illumina HiSeq platform mRNA gene expression, copy number variation (CNV) and clinical information of BLCA patients were downloaded from TCGA (Date: 09/26/2014) using TCGA assembler (29). MapSplice and RSEM algorithms were used for the short-read alignment and quantification of the mRNA sequencing data (Referred as RNASeqV2 in TCGA) (30, 31). The CNV of tumors were represented as normalized log ratio (base 2) between the tumor and normal reference. Gene level CNV was estimated as the mean of copy numbers of the genomic region of a gene using Bioconductor package DNAcopy (32) (retrieved by TCGA assembler directly). Patients with both gene expression and CNV data were included in the analysis (*n* = 308). GISTIC2.0 (33) whose number estimates of CNV gains/deletion in 24,776 unique genes were downloaded from TCGA firehouse (http://gdac.broadinstitute.org/; download date 03/09/2015). The GISTIC encodes homozygous deletions, heterozygous deletions, low-level gain and high-level amplifications as −2, −1, 1 and 2 respectively. The four types of events were counted for each of the patients. The total number of events was calculated by summing all four types of events.

Before applying MOGSA, minimal nonspecific filtering of low variance genes was performed on both data sets. Filtering was performed separately on each data type, and each could contain different biomolecules. Genes in RNAseq data were filtered to retain those with a total row sum greater than 300 (average RPKM > 300/308 in each patient) and median absolute deviation (MAD) greater than 0.1, which retained 14,692 unique genes (out of 20,531 genes). Minimal MAD based filtering removes low variant mRNAs with consistently low or high abundance across patients, which are unlikely to contribute information to a large-scale experiment. RNAseq data were log transformed (base 10) and median centered. We observed that most genes do not have CNV (log 2 CNV close to 0; supplemental Fig. S5). To exclude uninformative CNV data, CNVs with a standard deviation greater than the median were retained. A PCA of each individual data set of CNV and mRNA is shown in supplemental Fig. S6. In the scree plots of the first 10 eigenvalues, an elbow in each plot appears between 4–6 components suggesting this number of components is needed to capture most of the variance (supplemental Fig. S6), which was consistent with the reported molecular heterogeneity in these data.

##### BLCA Subtype Clustering

To determine the optimal number of components as input to MOGSA, we performed MOGSA on the BLCA mRNA gene expression and CNV data (*n* = 308) with a range of components from 1 to 12. For each gene-set in the GSS matrix, gene-sets were ranked by the number of tumors in which they were significantly regulated (either positive or negative GSS, *p* < 0.05), such that gene-sets that were significant in most tumors had the highest ranks. Most gene-sets were insignificant in all tumors and no gene-set was significant in all 308 tumors. For *p* < 0.05, we examined the 10, 20, 40, 100, 200, 500, and 1000 highest ranked gene-sets and examined the stability of gene-set ranking when additional components were included (supplemental Fig. S7). Increasing the number of components (from 1 to 5) increased the stability of gene-set lists, however there was little additional gain after five components in all cases (supplemental Fig. S7). In addition, we confirmed that these five components were not correlated with batch effects including TCGA batch ID, plates, shipping date or tissue source sites.

Next, consensus clustering was used (34, 35) to cluster the top five latent variables with Pearson correlation distance and Ward linkage for the inner loop clustering. Eighty percent of patients were used in the re-sampling step of clustering. Stability analysis showed there was no effect when different resampling proportions (50%, 60%, 70%, 80%, and 90%) were used in the inner and outer loop of consensus clustering (supplemental Fig. S8). Average agglomeration clustering was used in the final linkage (linkage for consensus matrix) (34).

##### Determining the Number of BLCA Clusters

Although consensus clustering analysis indicated high confidence in either two or three subtypes (supplemental Fig. S9), silhouette analysis (supplemental Fig. S9*E*) suggested three subtypes. A recent report highlighted limitations in consensus clustering (36), and therefore, in parallel, we also used the “prediction strength” algorithm, to discover the number of stable subtypes that can be predicted from the data (37). In the prediction strength method, all samples were assigned a “true” subtype label according to the clustering obtained from a given number of clusters. Then, the patients were divided into “training” and “testing” sets. The KNN classifier was used to classify the patients in the testing set. Cross-validation suggested that there is no obvious good choice of *K* (supplemental Fig. S10), and, therefore, we used a wide range of odd *K* from 1–17 (supplemental Fig. S11). For each test, the agreement in assignment between predicted and true labels were computed. The prediction strength was defined by the lowest proportion among all the subtypes. It indicates the similarity between the true and predicted labels and ranges from 0 to 1, where a value > 0.8 suggests a robust subtype classification (38). Therefore, the model with the greatest number of subtypes and prediction strength > 0.8 can be considered “optimal”. In this study, we performed 100 random separations of training and testing sets and the prediction strength of each randomization was calculated. The prediction strength analysis clearly supported three subtypes (supplemental Fig. S11). Therefore, using two independent approaches, we determined that the data (5 components of the integrated analysis) supported three BLCA molecular subtypes.

##### Sources of Gene-set Annotation

Molecular Signature Database MSigDB (version 4.0) (39) gene-sets included subsets C2 curated pathways, C3 motif pathways which included transcription factor target (TFT, *n* = 617) target gene-set and C5 gene ontology (GO) biological process (BP, *n* = 825), cellular component (CC, *n* = 233) and molecular function (MF, *n* = 396) terms. The pathway databases, Biocarta, KEGG and Reactome had 217, 186, and 674 gene-sets respectively. We excluded gene ontology terms that had more than 500 genes and less than 5 genes that mapped to features across all data sets. For example, in the BLCA analysis, gene-sets (1454 in total) were filtered to exclude those with less than 5 genes in a list of the concatenated features of CNV and mRNA data resulting in 1125 retained gene-sets.

##### Code Availability

The code used in this work is available on request.

## RESULTS

### 

#### 

##### Outline of MOGSA Algorithm

**Fig. 1.**
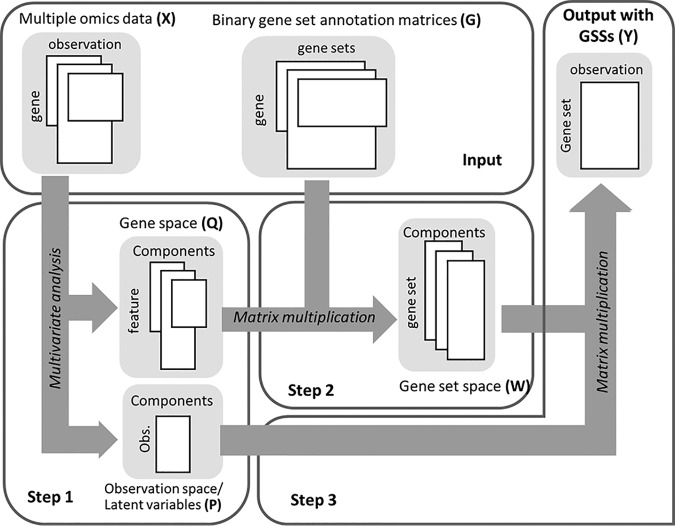
**Schematic representation of the MOGSA algorithm.** The algorithm requires pairs of matrices as input; multiple omics data matrices and corresponding gene-set (GS) annotation matrices. In step 1, the multiple matrices are analyzed using a multivariate analysis (MVA) method resulting in an observation space and gene space. Next, the gene-set annotation matrices are projected on the same space, and the resulting matrix contains the gene-set space. The last step is to reconstruct gene-set-observation by multiplying the observation and gene-set spaces.

MOGSA integrates multi-omics features measured on the same set of observations (*e.g.* cell lines, disease tissues) and learns multi-omics patterns of gene-sets that are significantly altered in these samples (Fig. 1). Omics studies can include multiple data matrices such as RNA sequencing counts of gene expression, abundance measurements of proteins, metabolites, DNA methylation, mutation or CNV data or other omics molecular data. The number of features may exceed the number of observations. We refer to genes, proteins or other biological molecules as features for simplicity below. Fig. 1 describes the three steps of the algorithm; Input omics data matrices must have matched observations but may have different and unmatched features. The number of features may exceed the number of observations. To map features to gene-sets, MOGSA also requires an incidence matrix of gene to gene-set membership associations for each data matrix, called “gene-set annotation matrix,” in which rows are features and columns are annotation vectors of gene-sets, a value of 1 indicates that a feature (*e.g.* gene) is a member of a gene-set. A feature may belong to multiple gene-sets simultaneously, that is a row sum may exceed 1.

In the first step, several (*k*) input data matrices are integrated using multiple factor analysis (MFA) (20). MFA is a multiple table extension of principal component analysis (PCA) that is well suited to integrating multiple omics data because it reduces high dimensional omics data to a relative small number of components that capture the most prominent correlated structure among different data sets (20, 40). To prevent data sets with more features or different scales dominating a MFA result, each data set is divided by the first eigenvalue of a decomposition of each individual data set. MFA generates matrices of latent variables (components) in observation (P) and feature (Q) space (Fig. 1). The number of components is typically less than the number of observations. We retain and examine the first few components as these represent most of the variance in the data. One can select non-contiguous components (to filter or omit one or more component). Approaches for choosing the number of components are discussed later. MOGSA makes use of an attractive property of MF approaches in that supplementary data such as gene-set information (*e.g.* Gene Ontology annotations) can be projected onto the observations space to aid interpretation (16, 17, 41). In the next step (step 2, Fig. 1) each gene-set annotation matrix (G_1..k_) is projected as additional information onto the gene-set space (Q_1..k_) generating a score for each gene-set in the same projected space (W_1..k_), where *k* is the number of data sets. In the final step (step 3, Fig. 1), MOGSA multiplies the latent variables of the observations (P) and latent variables of gene-sets (W_1..k_) to generate a matrix (Y) with a gene-set score (GSS) for each gene-set in each observation (Y).

A gene-set with a high GSS are driven by features that explain a large proportion of the global correlated information among data matrices. These features could be from one, many or all data matrices, and may be non-overlapping. For example a GSS of a gene-set with features A-H, could be driven by high levels of gene expression in genes A, B, C, and increased protein levels in proteins C, D, E and amplifications in copy number in gene H. The GSS matrix (Y) may be decomposed with respect to each data set (X) or latent variable space (P,Q) so that the contribution of each individual data set or component to the overall score can be evaluated (see Experimental Procedures for more details).

##### Multiple Omics GSA Has Greater Sensitivity to Identify Regulated Gene-sets

Biological pathways are regulated by a complex network of cellular molecules that can be measured using transcriptomics, phosphoproteomic, metabolomics, lipidomics or other molecular profiling methods. Popular ssGSA approaches, such as ssGSEA and ssGSVA are designed for analysis of a single data set. We postulated that there would be a greater power to detect gene-sets or cellular pathways if methods integrated more diverse data types. To this end, we compared the performance of MOGSA to ssGSA methods that were developed for the analysis of one data set including the widely used GSVA and ssGSEA and naïve matrix multiplication (NMM) methods (9, 10).

**Fig. 2.**
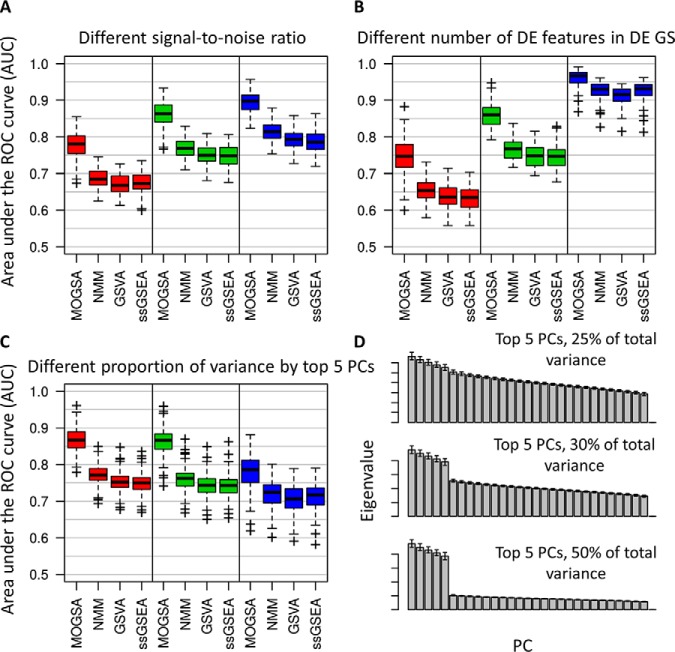
**Comparison of MOGSA with NMM, GSVA and ssGSEA.** The performance of each method was accessed by their ability to identify differentially expressed gene-sets over 100 simulations in every condition (as indicated by the area under the ROC curve; AUC). *A*, Comparison of GSA methods using data with different signal-to-noise ratios. From left to right, low (0.3), medium (0.5) or high (0.8) signal:noise ratio. *B*, Comparison of data with different number of differentially expressed (DE) genes in each of the DE gene-set. From left to right, 5, 10, and 25 of total 50 genes are differentially expressed in each of the three simulated data matrices if a gene-set is defined as DE gene-sets. *C*, Scree plots show representative eigenvalues in each of the conditions in (*D*). *D*, AUCs with different proportion of variance are capture by the top 5 components. From left to right, 25%, 30%, and 50% of total variance are captured. The darker bars represent the top 5 components.

Fig. 2 shows the performance of each method applied to 100 simulated data sets from non-overlapping gene-set simulation. In each data set, we simulated a study of 30 observations with three omics data types that measured 1000 features each (supplemental Fig. S1; see Experimental Procedures). Each feature was a member of one of the 20 gene-sets. Each gene-set had 50 genes. The observations were grouped into 6 clusters and each cluster had 5 differentially expressed (DE) gene-sets compared with the other observations. The triplets were analyzed by MOGSA directly, however, matrices were concatenated for NMM, GSVA and ssGSEA as these methods can only accept one matrix as input.

We expected that MOGSA would be especially powerful at identifying altered gene-sets in heterogeneous noisy data. That is because MOGSA uses only the top few most informative latent variables, thus omitting the signal of many features with little variance, which are potentially noise. Therefore, we explored the power of the methods to detect DE gene-sets when there was a strong or weak gene expression signal. First, we simulated increasing DEG signal-to-noise by changing the mean gene expression of DEGs in the cluster and secondly, we altered the number of DE genes in a DE gene-set (5, 10, and 25 genes). Specificity and sensitivity of the methods detecting the DE gene-sets were evaluated and the performances were summarized as the area under the receiver operating characteristic curve (AUC). As expected, the performance of all methods was better when the signal-to-noise ratio or the number of DE genes in DE gene-sets increased (Fig. 2*A* and 2*B*). MOGSA consistently outperformed the other methods and the difference was even more apparent when the signal-to-noise ratio was low or when there were few DE genes (5 or 10 of 50 genes) (Fig. 2*B*).

Next we compared the performance of each method using data with a simple or complex structure which reflect the complexity of phenotypes in a study. In data with a simple phenotype, a few components should easily capture most of the variances. However, in data with a complex phenotype, for example a heterogeneous tumor data set, with mixed histology, grade and response to treatment, there are many signals so that many latent variables may be required to capture even half of the variance. In the simulated data, observations grouped into six clusters, which could be captured by the first five components. Therefore, we simulated data such that the first 5 components captured 50%, 30%, or only 25% of the total variance (Fig. 2*C*). Again, MOGSA outperformed the other methods and was relatively robust to changes in the variance retained (Fig. 2*D*). The performance (AUC) of all methods decreased when greater variance was retained, which can be explained by higher intra-cluster correlation that leads to a lower signal-to-noise ratio in data sets (see Experimental Procedures). The same analyses were performed on the simulated data sets with overlapping gene-sets and led to similar results (supplemental Fig. S12).

Given the many fundamental differences between MOGSA and the other ssGSA methods, we repeated the simulations adjusting for technical aspects of the MOGSA approach that might give it an “unfair edge,” but these did little to improve the performance of the other methods. Because GSVA and ssGSEA were designed for analysis of single data sets, we compared the performance of GSVA and ssGSEA on a single data sets of the triplet compared with the concatenated triplet. Concatenating multiple data matrices neither improved nor decreased the performance compared with analysis of single data sets, which is most likely caused by the signal-to-noise ratio increased accordingly with concatenation (supplemental Fig. S13). In addition, because MFA weighs input matrices by their first singular value, we examined the effect of data set weighting on the other methods and found MOGSA still outperformed ssGSEA and GSVA when data matrices of the triplet were weighted before concatenation (supplemental Fig. S14*A*). To examine if *p* value distributions were well-calibrated and not inflated, we show a QQ-plot of MOGSA *p* values from analyses with no DE gene-sets in the three simulated matrices conformed to the null model (supplemental Fig. S14*B*).

##### MOGSA Provides An Easy Approach to Selectively Filter Or Enrich for Components

**Fig. 3.**
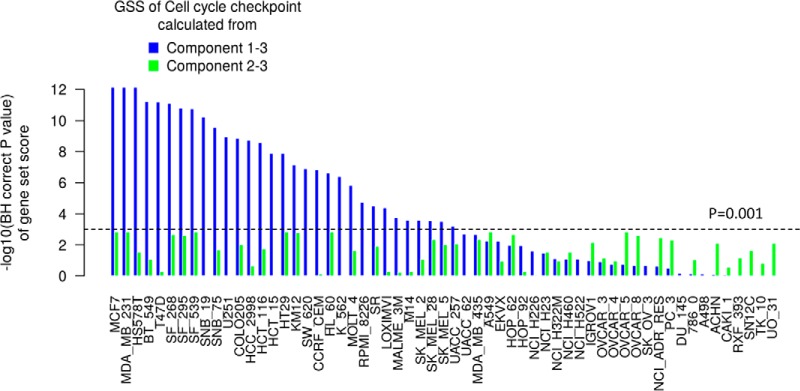
**Different gene-set scores by excluding components corresponding to unwanted variance.** The BH corrected *p* value of cell cycle checkpoint pathway (REACTOME) is increased, by excluding a component well correlated with cell doubling time (component 1). The corrected *p* value increased from <10^−12^ to >10^−3^ in MCF7 and MDA_MB_231 cells because component 1 is significantly associated with cell doubling time in these cell lines (see supplemental Fig. S13).

Large scale omics studies often have several sources of unwanted variance, because of technical batch effects (42) or unwanted biological sources, for example, tumor purity in bulk tumor studies (43) or cell cycle genes in single cell molecular studies (44). In our previous study, that integrated the proteome and transcriptome of 60 National Cancer Institute (NCI) cell lines, we observed that one of the top components in both data sets was correlated with cell doubling time (24). Cell doubling time in cell culture is a characteristic of the cell culture conditions and may not reflect the cell doubling time when cells are grown in more physiologically relevant three-dimensional culture models (45) or indeed those of tumors *in vivo*. We hypothesized that MOGSA would capture the doubling time effect as a component. A benefit of MF methods is that we can specifically exclude a component to remove the variance associated with it. In this case, by removing the component associated with doubling time (a feature of *in vitro* cell culture) we can potentially observe different gene-set scores which might be closer to *in vivo* biology. In our analysis, the significantly correlated structure between transcriptomic and proteomic data was captured in three components (see supplemental Fig. S2 and Experimental Procedures) and the first component which represented 6% variance was significantly correlated with cell doubling time (supplemental Fig. S15, Pearson Correlation *r* = 0.61, *p* = 3 × 10^−7^). Component 2 and 3 were not significantly correlated with cell doubling time (supplemental Fig. S15*A*). Component 2 was largely driven by the tumor type with leukemia and melanoma cell lines projected on the positive and negative ends respectively (supplemental Fig. S15*B*). Excluding the first component from score computations, removed the effect of cell doubling time (supplemental Fig. S16), generating alternative gene-set scores. For example, the gene-set score (GSS) for cell cycle checkpoint was significant, after adjusting for multiple testing using the Benjamini Hochberg (BH) method (BH corrected *p* < 0.001) in 31 cell lines, and after excluding the first component, was no significant in any of the cell lines (Fig. 3).

A stability analysis was performed to evaluate the robustness of components when cell lines were iteratively excluded (leave-one-out analysis) or between 10 and 50% features were randomly excluded from both data sets. There was a high correlation between the components in the original (*n* = 59) and in leave-one-out analyses of cell lines, indicating that MOGSA components are robust to small perturbation in samples (the average correlation was 0.984, 0.979, and 0.990 for the first three components, respectively). The first three components in analyses where up to 50% of features were excluded each had a correlation greater than 0.99 with the original analysis. (See Supplementary Methods Stability analysis of MOGSA components; supplemental Table S1 and supplemental Fig. S17).

##### Application of MOGSA to the Interpretation of Stem Cell mRNA and Proteomics Data

Many multiple omics studies have few samples and are performed to address targeted research questions in individual research laboratories. To demonstrate the application of MOGSA to a small data set with few samples, we applied MOGSA to study profiles of mRNA (*n* = 10,961), protein (*n* = 5817) and phosphorylation sites of proteins (*n* = 7912) of four cell lines - two embryonic stem cell lines (ESC; H1 and H9), one induced pluripotent cell line (iPSC; DF19.7) and a fibroblast cell line (newborn foreskin fibroblast; NFF). Induced pluripotent stem cells (iPSC) are adult cells that have been reprogrammed to be more like embryonic stem cells (ESC) and have great potential in the field of regenerative medicine. These cells express ESC markers and can differentiate into different cell types (26). Induced pluripotent cells are often derived from NFF cells.

MFA recapitulated the PCA of the individual data sets (supplemental Fig. S18, S4). The three data sets contributed similar variance in the integrated analysis, as indicated by the weights of each data set in MFA. The variance of the first PCs were 0.24, 0.26, and 0.26 for the transcriptome, proteome and phospho-proteome data set respectively. Most of the variance was captured in the first component and it discriminated between NFF and other cell lines. The variance of the molecular differences between the ESC cells (captured by the second component) was greater than the difference between ESC and iPSC cell lines (component 3; supplemental Fig. S18).

**Fig. 4.**
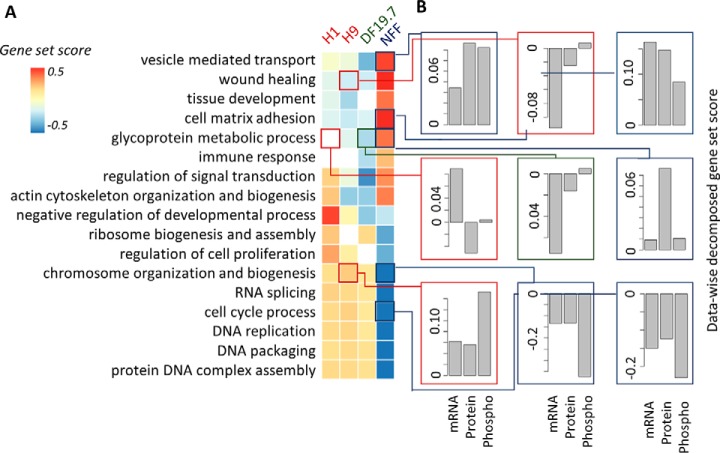
**integrative gene-set analysis of iPS ES 4-plex data.** *A*, Heatmap showing the gene-set score (GSS) for significantly regulated gene-sets in the cell lines. The white colored blocks/cells indicate that the change of gene-sets are non-significant (BH corrected *p* > 0.01). *B*, Data-wise decomposition of the GSS for some of the gene-sets. The contribution of each of the data is represent by a bar. The *y*-axis is the data-wise decomposed gene-set score.

MOGSA of gene ontology (GO) biological processes (BP) discovered 228 GO terms (out of 825) that had significant up or downregulated gene-set scores (GSSs) in at least one cell line (BH corrected *p* < 0.01). These 228 GO terms grouped into 21 broad categories, when overlaps of gene membership to GO terms was reduced using hierarchical clustering analysis (Hamming distance and complete linkage; supplemental Table S2). GSS of representative GO terms from each category are shown in Fig. 4*A*. Biological processes associated with more differentiated cell types were associated with the NFF cells and included upregulation of vesicle-mediated transport, immune related responses and cell adhesion. In contrast, cell proliferation GO terms such DNA replication, and cell cycle processes had significantly higher GGS in the highly proliferative stem cell lines. These results are consistent with previous findings (26).

In the integrative analysis of multiple omics data, it is important to evaluate the relative contribution (either concordant or discrepant) of each data set to the overall GSS. Data-wise decomposition of the GSSs (see Supplementary Methods) are shown in Fig. 4*B*. The three data sets had concordant contributions to most of the GO terms, including vesicle mediate transport, cell matrix adhesion, cell cycle processes in NFF line; chromosome organization and biogenesis in H9 and NFF cell lines. However, we also observed differences in the contribution of mRNA, proteins and phosphoprotein data to the GSS, for example chromosome organization and biogenesis had significant positive GSS in the stem cells and significant negative GSS in the NFF cells and were driven by differences in the phosphorylation data. Another case where the mRNA and protein were incongruent is the GO class “glycoprotein metabolic process.” The significant (BH corrected *p* < 0.01) GSSs of this term are 0.097, −0.086 and −0.053 in NFF, iPSC and H9 cells respectively. Upregulation in NFF mainly reflected upregulation on the protein level. However, downregulation in iPSC DF19.7 cells was because of low expression of related mRNAs. The GO term “wound healing” has previously been shown to be differentially upregulated in fibroblast NFF cells compared with ESC (26). Consistently, we also found wound healing to be upregulated in NFF compared with ESC; the significant (BH corrected *p* < 0.01) GSSs for wound healing were 0.36, −0.13, −0.13, and −0.09 for NFF, iPSC, H9, and H1 cells respectively (supplemental Table S2). Downregulation of wound healing in H9 cell line was dominated by mRNA data, and the two proteomics data sets contributed little to the negative GSS. In contrast to previous studies (26). We additionally observed significant differences in wound healing between iPSC and ESC. Thus, MOGSA may have greater sensitivity to detect gene-sets that have consistent but subtle changes in multiple data sets. Importantly, the contribution of individual data sets to gene-sets could be evaluated by the decomposition of GSS, thus facilitating hypothesis generation and design of subsequent experiments.

##### Application of MOGSA to Molecular Subtype Discovery in Bladder Cancer

The aim of large multi-omics studies is frequently to discover molecular subtypes in cohorts of heterogenous samples, which is achieved through unsupervised cluster analysis. MOGSA can transform multiple molecular profiling data (mRNA, CNV, protein) to a gene-set by sample matrix to increase the power of subtype discovery in these applications.

Recent efforts have focused on identifying molecular subtypes in BLCA to tailor therapy and improve outcomes. BLCA is a molecularly heterogeneous cancer with between 2 and 5 molecular subtypes (reviewed by (46, 47)). Briefly, Sjödahl *et al.* first defined five major subtypes termed urobasal A (UroA), UroB, genomically unstable (GU), squamous cell carcinoma-like (SCCL) and “infiltrated” (38). The TCGA study defined four expression clusters (I-IV) (46). The two subtype model consists of basal-like and luminal subtypes (48) which was extended by Choi *et al.* who defined a “p53-like” luminal subtype apart from basal-like and luminal subtypes (49). Therefore, to exemplify the application of MOGSA in large multi-omics studies, we applied it to a cohort of 308 muscle invasive urothelial bladder cancer (BLCA) patients (obtained as part of the TCGA project), where 12,447 CNVs and 14,719 mRNAs were measured to learn an integrative subtype model of BLCA (see Experimental Procedures).

**Fig. 5.**
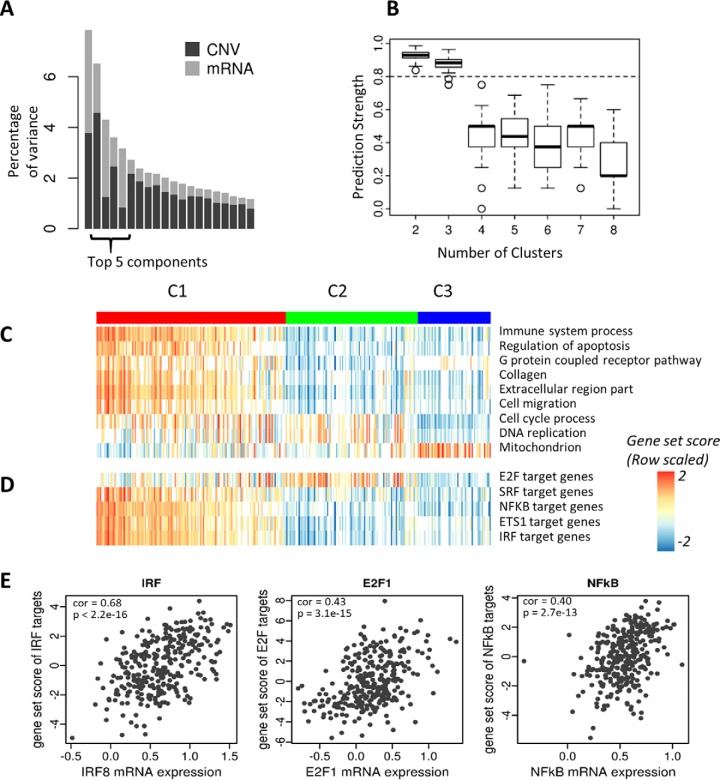
**Data integration using MOGSA and integrative subtype defined by latent variables.** *A*, Bar plot showing the eigenvalues of components defined by MFA. The top 5 components were selected in the analysis. *B*, Prediction strength was used to evaluate the robustness of classification into two to eight subtypes. The boxplot shows the prediction strength of 100 randomizations. Two and three are relative robust subtype models (prediction strength > 0.8). *C*, Gene ontology (GO) and transcriptional target (TFT) gene-sets annotation of tumors. Heatmap showing the GSSs for selected gene-sets. The gene-sets “immune-related, apoptosis, G protein receptor, collagen, extracellular region and cell migration” are strong in the C1 (basal-like) subtype, whereas the mitochondrial related gene-sets are over represented in the C3 (luminal A-like) subtype of tumors. *D*, The most significant transcriptional factor (TF) target gene-sets. The gene-set scores suggest that 4 out of the 5 TFs are hyperactive in the C1 subtype, except E2F family is active in the C2 subtype of cancer. The white spaces in (*A*) and (*B*) denote non-significant GSSs. *E*, The scatter plots display the correlation between gene-set scores and the mRNA level of selected TFs. The expression of selected TFs is significantly correlated with their gene-set scores (also see supplemental Fig. S21).

In our analysis, the top five MFA components captured a quarter of the total variance and were not dominated by either CNV or mRNA (CNV 50.6%, mRNA 49.4%; Fig. 5*A*). We performed extensive analyses to confirm that 5 components captured enough variance and was the optimal number of components as input to MOGSA (see “Experimental procedures”). Similar to the stability analysis of NCI60 data, these components were robust and stable when subsets of patients (22 iterations where 14/308 patients were excluded each time) or random features (between 10% to 50%) were removed from the data sets (See Supplementary Methods and supplemental Table S1). We examined 1,125 gene-sets using MOGSA in every individual patient. The number of significant MOGSA gene-sets per patient (BH corrected *p* < 0.05) ranged from 46 to 338 (supplemental Fig. S19). Each patient had both positive (up) and negative gene-set scores (GSSs). We focused on the 84 gene-sets that were significantly regulated (positive or negative GSS scores, BH corrected *p* < 0.05) in more than half of the patients (supplemental Table S3 and supplemental Fig. S20). Cluster analysis of the GSS matrix (84 selected gene-sets × 308 tumors) revealed three clusters of gene-sets.

##### Validation of the Number of BLCA Clusters

To identify the number of robust BLCA clusters, we applied several cluster analyses to the five components (which were input to MOGSA) and tested how many clusters were supported by the data. We applied rigorous cluster robustness analysis using methods including prediction strength and confirmed that the data supported three subtypes (Fig. 5*B*), the final three subtype model was learned from consensus clustering (34) of the five components and the robustness of the three BLCA subtype model was extensively validated (see Experimental Procedures). The three integrative BLCA subtypes consisted of two larger subtypes C1, C2 containing 148 and 103 patients respectively, and a robust smaller group C3 with 57 patients (Fig. 5*C*).

The clusters obtained by hierarchical clustering analysis of the 84 gene-sets scores from MOGSA were consistent with the three clusters obtained by consensus clustering of the five components. A large cluster of 54 gene-sets had positive GSS scores in C1 but negative scores in C2 or C3. Two smaller clusters of gene-sets of 17 and 10 gene-sets had positive GSS scores in C2 and C3 respectively (supplemental Fig. S20).

We compared the overlap between the integrative BLCA subtypes and with BLCA subtypes reported in previous studies (46) (supplemental Table S4, supplemental Fig. S21). Subtypes C2 and C3 were like the Damrauer luminal subtype (48). But the C3 subtype contained more low-grade tumors and showed a strong overlap with the UroA subtype of the Sjödahl study and type I of the TCGA subtype model. Subtype C2 tumors overlapped with the genomically unstable subtype defined by Sjödahl (supplemental Table S4). Accordingly, we observed higher mutation rates in the C2 patients (supplemental Fig. S22). The integrative subtype C1 included a high number of patients in the type III and IV of the TCGA subtypes, the infiltrated and SCCL subtypes of the Sjödahl study (38) and the basal-like subtype identified by Damrauer (BH corrected *p* value < 0.05, (supplemental Table S4) (48).

##### Gene-sets Enriched in the Three BLCA Molecular Subtypes

The large number of the C1 basal-like/SCC-like BLCA gene-sets (31/51) were associated with “immune response,” which supports associations between immune regulation and the basal-like cluster that have been previously reported (38) The most significant gene-sets, “immune response” and “immune system process” had significant positive or negative GSS in 259 and 254 of 308 patients respectively (supplemental Table S3). The median GSS for the gene-set “immune system process” was 0.82, −0.75, −0.61 in C1, C2, and C3 respectively (Fig. 5*C* and supplemental Fig. S23) indicating that immune related processes have high gene expression or copy number amplification in the C1 subtype and much lower in C2 and C3.

The remaining 20/51 gene-sets in the C1 cluster of gene-sets reflected “extracellular”, function, cell morphogenesis, migration and muscle cell development, “apoptosis” (2 gene-sets), and “G protein coupled receptor” (6 gene-sets) (Fig. 5*C* and supplemental Fig. S23) and EMT related gene-sets (supplemental Fig. S24), which is consistent with reports that the Basal-like subtype has more muscle-invasive and metastatic disease at presentation (38). Gene-sets related to “cell cycle” (9 gene-sets) and “DNA repair and chromosome related” (7 gene-sets) had high GSS in luminal gnomically unstable C2 (50) (and some C1) and “mitochondrion” (4 gene-sets) in C3 (Fig. 5*C* and supplemental Fig. S23). The mitochondrial component has been described in bladder cancer and other cancers previously (50, 51) and our study particularly associated this function with C3 low-grade papillary-like subtype in BLCA.

##### Discovery of Transcription Factors That Might Regulate Gene Expression in BLCA Subtypes

To identify transcription factors (TF) that may regulate gene expression in the three tumor subtypes, we used transcriptional factor target (TFT) gene-sets to annotate the tumors. Like the selection of GO terms, we focused on TFT gene-sets with more than 154 significant GSSs across 308 patients (BH corrected *p* < 0.05; supplemental Table S3). The GSSs of the E2F family target gene-set were significantly different in most of the tumors and are particularly low for the C3 tumors. Four identified TFs that were highly elevated in the C1 subtype; *MADS* (*MCM1*, Agamous, Deficiens, and *SRF*) box superfamily member, *SRF* and several TFs associated with transactivation of cytokine and chemokine genes, including *NFkB1*, *ETS1,* and *IRF1* (Fig. 5*D*). The genes exhibiting the largest gene influential score (GIS; see Supplementary Methods) in the *IRF1* and *NFkB1* target gene-sets included *ACTN1*, *CXorf21*, *ICAM1*, *MSN*, *TNFSF13B*, *IL12RB1,* and *CDK6* (supplemental Table S5). Further, we examined the correlations between GSSs and the mRNA expression. All five TFs showed that the TF mRNA and GSSs are significantly correlated (Fig. 5*E* and supplemental Fig. S25).

##### The Gene Influence Score (GIS scores) - Evaluating Influence of Individual Genes in Gene-set Scores

In studies trying to define molecular biomarkers, a single or small number of genes or proteins are often preferred as opposed to a gene-set, which may contain hundreds of genes. To evaluate the importance of individual features in a gene-set, we calculated a gene influential score (GIS) using a leave-one-out procedure (see Supplementary Methods). The maximum GIS value for a gene in a gene-set is 1, which indicates that this gene contributes a high proportion of variance to the overall variance of the GSSs. A GIS close to 1 often suggests a high correlation between the gene expression value and GSS. Gene influential scores of the gene-set immune system process in BLCA suggested that the top ranked genes included *ITGB2*, *SPI1*, *DOCK2*, *LILRB2,* and *LAT2*. Other highly ranked genes included drug target genes such as *CD4*, *IL6*, the interferon induced proteins *IFITM2* and *IFITM3* and the G protein coupled receptors *GPR183* and *CMKLR1* (supplemental Table S6). Moreover, the C3 subtype tumors had higher GSSs in mitochondrial related gene-sets and lower expression of genes related to cell cycle process and DNA replication. GIS analysis suggested that two families of genes, NADH dehydrogenases (NDUFs) and mitochondrial ribosomal proteins (*ABCC1*/*MRP*) influenced the mitochondrial proteins (supplemental Table S6).

##### Decomposing GSS Scores by Data Type

**Fig. 6.**
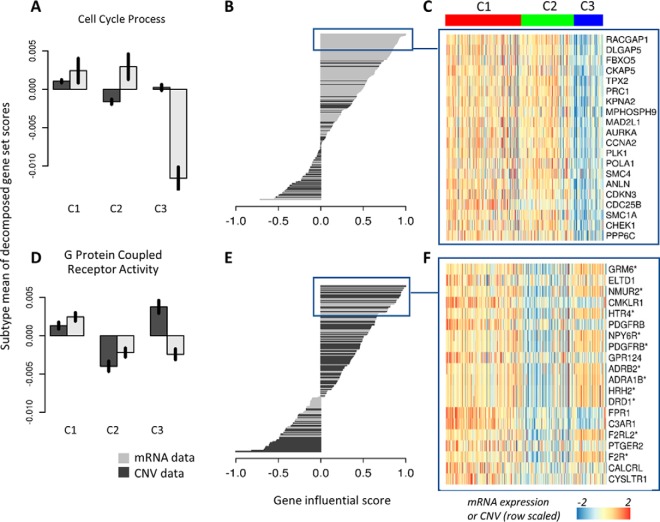
**CNV and mRNA data contribute unequally to defining subtype and gene-set scores.** *A*, Data-wise decomposition of gene-set scores for “cell cycle process.” The bar plot shows the normalized mean of data-wise decomposed GSSs in each subtype (the black vertical line on the bars show the 95% confidence interval of the mean). *B*, The bar plot shows the gene influential scores (GISs) of genes in the “cell cycle process” gene-sets. The expression of the top 30 most influential genes in the gene-set are shown in (*C*). *D–F*, Same as (*A–C*) for “G protein couple receptor activity.” Gene names in (*F*) with asterisks indicate genes from CNV data.

We decomposed the GSSs with respect to the data sets and performed a gene influential score (GIS) analysis to learn the mRNA and CNV features that influenced each GSS. For example, Fig. 6*A* shows a GIS analysis of the GSS “cell cycle process,” where we found that mRNA expression strongly influenced the GSS, particularly the low GSS of the C3 subtype patients. The top 30 most influential genes were all observed in mRNA expression data (Fig. 6*B*), including *RACGAP1*, *DLGAP5*, *FBXO5*, *AURKA*, *KERA* (*CNA2*), and *CDKN3* (Fig. 6*C*). Indeed, data-wise decomposition of GSS identified several GSSs that were driven by the mRNA data, including the immune system process, DNA replication and mitochondrion gene-set (supplemental Fig. S26). By contrast, both CNV and mRNA data influenced the gene-set “G protein coupled receptor activity” (Fig. 6*D*) and the most influential genes (the GIS analysis) included both mRNA and CNV data (Fig. 6*E*), and these differentiated the C3 subtype (Fig. 6*F*). The features that scored highly in the GSS “G protein couple receptor activity” included CNV of *GRM6*, *NMUR2* and adrenergic receptors, and the gene expression of *ADGRL4* (*ELTD1*), *CMKLR1* (Fig. 6*F*). In this gene-set, the cancer driver gene and drug target *PDGFRB* were identified on both CNV and mRNA levels.

##### Computational Performance

We applied MOGSA to various data set sizes and compared its computational efficiency to other methods. It performs comparably or marginally slower than ssGSEA, but it is faster than GSVA (supplemental Fig. S27) and much faster than coGAPS (data not shown as it failed to compute on larger data sets in our studies).

## DISCUSSION

In this manuscript, we introduced a new approach for multi-omics ssGSA, termed MOGSA that enables the discovery of *e.g.* biological pathways with correlated profiles across multiple complex data sets. MOGSA uses tensor MF or multivariate latent variable analysis to explore correlated global variance structure across data sets and then extracts the gene-sets or pathways with the highest variance that are most strongly associated with this correlated structure across observations. By combining multiple data types, we can compensate for missing or unreliable information in any single data type so we may find gene-sets that cannot be detected by single omics data analysis alone (15).

MOGSA is fundamentally different from other gene-set enrichment analysis methods which use a 'within observation summarization' such as the mean or median of gene expression of genes in a gene-set (9, 10, 40). MOGSA has several unique characteristics that make it well suited for generating integrated multi-omics gene-set scores. First, MOGSA uses MFA, a multitable extension of PCA to reduce the complexity of the original data by transforming high dimensional data to a low dimensional representation of the data on a few orthogonal components (latent variables). The components with the highest eigenvalues capture the most prominent or variant structure that is shared among the different data sets. Keeping the first few components (with high variance) and excluding lower ranked components that may be associated with noise or artifacts (52, 53) may increase the signal-to-noise ratio and sensitivity of data analysis. In MOGSA, the entire set of features from each platform is decomposed onto a lower dimension space. The linear combination of feature loadings is used in the calculation of the gene-set scores. Features that contribute low variance contribute little to the score and thus the dimension reduction within MOGSA provides an intrinsic filtering of noise. The advantages of intrinsic variance filtering of features can be clearly seen when we applied MOGSA to simulated data. Second, data integration of features is achieved at the gene-sets level rather than scoring individual features. This greatly facilitates the biological interpretation among multiple integrated data sets. There is no requirement to pre-filter features in a study or map features from different data sets to a set of common genes. Therefore, MOGSA can be used to compare technology platforms that have different or missing features.

There is great potential for applying multitable unsupervised GSA approaches for discovery of new subtypes and pathways in integrated data analysis of complex diseases such as cancer. In this study, we applied MOGSA in combination with clustering analysis. Recent studies comparing algorithms clustering multi-omics data have confirmed a good performance of matrix-factorization-based algorithm in terms of speed and cluster accuracy (54–56). This may because these approaches consider the global variance in the data and as such are complementary to hierarchical or k-means clustering approaches which focus on the pair-wise distance between observations (53, 57–59).

The number of components is an important input parameter to consider when applying MOGSA to gene-set analysis or cluster discovery. Like PCA, the optimal number of MFA components may be assessed by examining the variance associated with each component. The first component will capture most variance and the variance associated with subsequent component decreases monotonically. Scree plots (Fig. 2*D*, 5*A*) may be used to visualize if there is an elbow point in the eigenvalues, allowing one to select the components before the elbow point. Alternatively, one may select the number of components that capture a certain proportion of variance (50%, 70%, etc.). In addition, one may include components that are of biological interest. For example, in the iPS ES example, there is a clear biological meaning in the third component (ES *versus* iPS cell line). Permutation analysis is a more objective approach to select components in PCA and factor analysis (60). We used a permutation-based method in MOGSA of NCI60 data set, *i.e.* the samples in each omics data set are randomly permutated multiple times and a null distribution of variance associated with each component is calculated from these permutated samples. This method can be used to identify the components representing common structures in multiple omics data sets and requires the least subjective interpretation. Therefore, we implemented this in the MOGSA package.

In the analysis of the BLCA data, we examined components 1 to 12 and showed that there is little gain of information once a minimum number of components with high variance are included (Fig. 6*B*). Users should also consider that the variance of retained components should not be dominated by one or a few data sets. To facilitate biological interpretation of components, the GSS could be decomposed regarding components. In the BLCA example, the second and forth component are largely contributed by CNV, whereas mRNA is more important in defining the third and fifth components. Including five components ensured that both data sets contributed relatively similar variance to the total gene-set score.

An issue might arise with latent variables analysis if components with large variance capture information unrelated to biological variance such as technical artifacts or batch effects. In practice, this is rare in MFA, because it focuses on components that capture global correlation among all data sets. Often batch effects are specific to a platform and thus a component that captures information that is entirely uncorrelated to the global structure will be omitted from the set of highly variant integrated components. Decomposition of gene-sets by components makes it is easy to remove unwanted variance, and gene-set scores calculated from selected components, reflecting variance of interest, may lead to the better interpretation of interesting biology. However, it is still wise to perform careful batch effect control on individual data sets. To this end, Surrogate Variable Analysis (SVA) (14) can be used to detect unwanted variance in a single data set where the samples groups are known a priori (*e.g.* disease *versus* normal tissues). However, SVA is not optimal in experiments like the BLCA where predefined groups are unknown. In multi-omics experiments, a data set with platform-specific batch effects can be detected by calculating pair-wise RV coefficients (61) between data sets. RV coefficients, ranging from 0 (low similarity) to 1 (high similarity), are a generalization of Pearson correlation coefficients used to quantify the similarity between two matrices. A data set with consistently lower RV coefficients when compared with other data sets may contain some data set-specific biology or technology batch effect. To include such a data set in MOGSA, the weight or relative importance of that data set to the integrative analysis can be reduced or normalized using STATIS (62) (which is implemented in MOGSA). STATIS normalizes data sets to give a greater weight to a data set with variance close to most data sets and a lower weight to a data set deviating from the majority. As a result, the resulting components are more likely represent all data sets rather than being driven by one outlier data set.

When batch effect or other unwanted variance are detected, MOGSA may calculate gene-set scores based on selected components to enrich for specific patterns or exclude attributes. For example, we excluded the first component in the NCI60 cell line data which was associated with cell doubling time. Another consideration when applying MOGSA is that it is most efficient in detecting gene-sets that have broad correlation patterns among data types. It may fail to discover gene-sets with few genes, particularly if they had low variances on the selected components.

MOGSA relies on a linear multivariate analysis method, that means that the resulting components and feature contributions capture linear correlation structure between multiple omics data sets. A potential nonlinear structure may not be captured by MOGSA. However, linear approximation of omics data sets is computational efficient and has been successfully applied to find prominent structure in noisy omics data (15, 16). T-Distributed Stochastic Neighbor Embedding (t-SNE) is a nonlinear dimension reduction method widely used in omics data analysis (63). It preserves polynomial structure in omics data sets and projects the nonlinear variance onto a lower dimensional space. However, t-SNE is not well suited for GSA, because it uses a stochastic procedure and contributions of individual features cannot be explicitly assessed. A more promising extension of MOGSA to account for nonlinear structure is to use a kernel function in the MFA step (64). However, extending MOGSA to nonlinear kernels will considerably increase computational time because it dramatically expands the parameter space that needs to be optimized. Therefore, combining nonlinear kernels and MFA must be further evaluated in omics data integration.

Sample size is also a consideration when applying MOGSA. Although MOGSA is a single sample GSA method, it should not be used when only one sample is measured, because MOGSA normalizes measured values across multiple samples (feature-wise normalization). This is like ssGSEA and GSVA which use multiple samples to calculate a cumulative density function. In addition, MOGSA should be applied with caution when the sample size is too small (*i.e.* n < 3) because the feature-wise normalization process tends to be unstable in these cases. The current implementation of MOGSA employs classical MF and is restricted to studies where the samples are measured in all data sets, but missing data would be better accommodated if it were extended to use a Bayesian MF approach, such a group factor analysis, which has been successfully applied to multi-omics data analysis (40).

Although the matrix factorization step does not require “ID mapping” in MOGSA, it is still required in the gene-set annotation step. Hence, the features included in gene-set scores calculations are feature annotated to gene-set and thus are limited by the incompleteness and inaccuracy of gene-set annotation databases. Currently most databases only include information on the gene or protein level and do not include transcript level, site level of PTMs or metabolites. Therefore, we used protein level annotation to annotate phospho-sites in this study. When more annotations are available, MOGSA can easily incorporate them through defining gene-set annotation matrices based on different levels of information. This would allow recent gene-set collections at the PTM level (65) to be used. Equally, MOGSA can learn or predict the function of unknown or poorly annotated biomolecules using by “guilt-by-association” inference, which could be used to extend gene-set databases to multi-omics complexity.

Finally, MOGSA is computationally efficient when applied to large omics integrative analysis of mRNA and CNV data of over 10,000 tumors. Open source, well documented code is provided in the Bioconductor package MOGSA which includes a detailed vignette tutorial and example data sets. Although we do not include examples applied to multi-omics single cell sequencing technologies that provide simultaneous measurement of transcriptomes and protein markers expressed in the same cell, *e.g.* CITE-seq or REAP-seq (4, 5), MOGSA can be applied to integrating, interpreting and generating biological hypothesis from complex high dimensional data sets from bulk sequencing or single cell technologies.

## Data Availability

NCI60 proteomics data (http://129.187.44.58:7070/NCI60/ or https://www.ebi.ac.uk/pride/archive/projects/PXD005946). NCI60 transcriptomics data (https://discover.nci.nih.gov/cellminer/ (RNA: 5 Platform Gene Transcript (average z score)). IPS ES 4-plex data: supplemental Table S1, S2, and S5 from http://scor.chem.wisc.edu/data.php. BLCA GISTIC data (https://gdac.broadinstitute.org/ (Bladder urothelial carcinoma)). BLCA CNV and mRNA data (https://portal.gdc.cancer.gov/).

## Supplementary Material

supplemental Fig. S13

SupplementaryInfo

table S1

Table S2

Table S3

Table S4

Table S5

Table S6

## Footnotes

^1^ The abbreviations used are:

GSA: gene-set analysis
ANOVA: analysis of variance
AUC: area under the receiver operating characteristic curve
BLCA: bladder cancer
BP: biological process
CC: cellular component
CCA: canonical correlation analysis
CIA: co-inertia analysis
CLT: central limited theorem
CPTAC: Clinical Proteomic Tumor Analysis Consortium
DE: differentially expressed
DEGS: differentially expressed gene-set
EMT: Epithelial to mesenchymal transition
GIS: gene influential score
GO: gene ontology
GS: gene-set
GSEA: gene-set enrichment analysis
GSS: gene-set score
MAD: median absolute deviation
MCIA: multiple co-inertia analysis
MF: molecular function
MF: matrix factorization
MFA: multiple factorial analysis
MVA: multivariate analysis
NMM: naïve matrix multiplication
PCA: principal component analysis
ROC: Receiver operating characteristic
SVD: singular value decomposition
TCGA: the cancer genome atlas
TF: transcriptional factor
TFT: transcriptional factor target
t-SNE: t-Distributed Stochastic Neighbor Embedding.
