# Supplementary material for: MOGSA: Integrative Single Sample Gene-set Analysis of Multiple Omics Data
Source: Mol Cell Proteomics. 2019 Jun 26;18(8 Suppl 1):S153–68. doi: 10.1074/mcp.TIR118.001251 (PMC6692785; doi:10.1074/mcp.TIR118.001251)
Supplement: supplemental Fig. S13 [file TIR118.001251_index.html]

Supplement to MOGSA: integrative single sample gene-set analysis of multiple omics data | Molecular & Cellular Proteomics

## Supplemental Data

- SupplementaryInfo - supplementary methods and figures
- table S1 - the results of component stability analysis for the NCI60 and the BLCA studies.
- Table S2 - the gene-set score (GSS) matrix of Gene ontology (GO) for iPS ES 4-plex data.
- Table S3 - the gene-set score (GSS) matrix of Gene ontology (GO) and transcriptional factor target (TFT) gene-set with more than 200 significant GSSs for BLCA data.
- Table S4 - The Chi square test of association between integrative subtypes and previously published subtypes.
- Table S5 - the gene influential score (GIS) for selected transcriptional factor gene-sets. The document contains GIS analysis for 2 gene-sets.
- Table S6 - the gene influential score (GIS) for selected gene-sets (from Gene Ontology). The document contains GIS analysis for 9 gene-sets.
